# Immunoglobulin Therapy in a Patient With Severe Chikungunya Fever and Vesiculobullous Lesions

**DOI:** 10.3389/fimmu.2019.01498

**Published:** 2019-07-02

**Authors:** Ana Isabel V. Fernandes, Joelma R. Souza, Adriano R. Silva, Sara B. S. C. Cruz, Lúcio R. C. Castellano

**Affiliations:** ^1^Human Immunology Research and Education Group-GEPIH, Escola Técnica de Saúde da UFPB, Universidade Federal da Paraíba, João Pessoa, Brazil; ^2^Division for Infectious and Parasitic Diseases, Hospital Universitário Lauro Wanderley, Universidade Federal da Paraíba, João Pessoa, Brazil; ^3^Department of Physiology and Pathology, Universidade Federal da Paraíba, João Pessoa, Brazil

**Keywords:** Chikungunya fever, therapeutics, intravenous antibodies, flebogamma DIF, intensive care

## Abstract

Chikungunya virus (CHIKV) is an emerging arbovirus whose transmission has already been reported in several countries. Although the majority of individuals acutely infected with CHIKV appear to become asymptomatic, reports showing the occurrence of atypical and severe forms of the disease are increasing. Among them, the neurological and skin manifestations require medical attention. Treatment of CHIKV infection is almost symptomatic. In this sense, we report the case of a 56-years-old man who presented fever, headaches, paresthesia and pain in the right arm with visible red spots on the skin starting 30 days before Hospital admission. Tests determined Chikungunya infection and excluded other co-morbidities. Disease evolved with edema in hands and feet and extensive hemorrhagic bullous lesions on the skin of upper and lower limbs. Variations in hematological counts associated with liver dysfunction determined this patient's admission to the Intensive Care Unit. Then, he received intravenous antibiotic and immunoglobulin therapy (400 mg/Kg/day for the period of 5 days) with total recovery from the lesions after 10 days of follow-up. A general improvement in blood cell count and successful wound healing was observed. After discharge, no other clinical sign of the disease was reported until nowadays. This case reports for the first time the successful administration of intravenous immunoglobulin therapy to a patient with severe atypical dermatological form of Chikungunya Fever without any associated comorbidity.

## Background

Chikungunya virus (CHIKV) infection is an acute febrile illness, accompanied by cutaneous rash and joint pains (1). Atypical and severe forms of the disease were responsible for 10.6% of deaths during an outbreak in Central America (2, 3). Recently, a series of four fatal cases presenting atypical CHIKV infection have been reported in the Brazilian state of Paraíba (4). Atypical skin manifestations associated with CHIKV infection are of great importance especially in infants and elderly patients (5, 6). Rash, pigmentation, and erythematous maculopapular rash affecting the trunk, limb and face are the most prevalent mucocutaneous manifestations of CHIKV infection (7, 8).

For both typical or atypical CHIKV infection, treatment based on antivirals, and rehydration is not successful in some cases, thus requiring development of new therapeutic strategies (9). Despite the increasing number of atypical and fatal CHIKV cases and their importance worldwide, many drugs that have shown promise *in vitro* remain unproven *in vivo* (10).

Recently, some reports demonstrated that the administration of human antibodies to treat CHIKV infection serves as an alternative therapy to treat neurologically severe forms of the disease (9, 11–13). The Intravenous immunoglobulin IVIG-Flebogamma® is a blood product usually prepared from the serum of 1,000 donors per batch. It is constituted as Normal Human Immunoglobulin (active substance) and contains the IgG antibodies present in the normal population while its subclass distribution of immunoglobulin G is almost proportional to that of functional human plasma (66.6% IgG1, 28.5% IgG2, 2.7% IgG3, and 2.2% IgG4). It is the treatment of choice for patients with antibody deficiencies usually employed at a replacement dose, but, when at higher doses, it might be used as an immunomodulatory agent in immune and inflammatory disorders, with special attention to dermatological diseases (14).

We report herein a case of a CHIKV-infected patient presenting atypical skin manifestation treated for the first time with intravenous immunoglobulin therapy.

## Case Report

**Figure 1:**
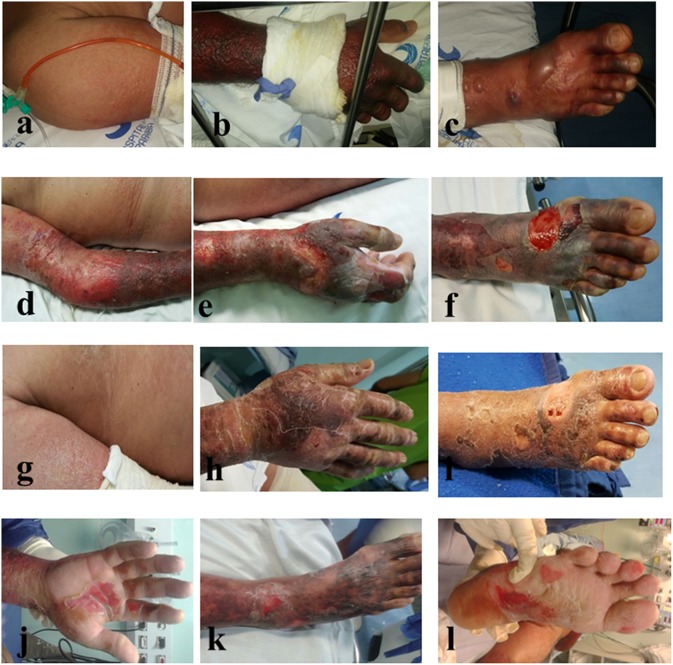
Skin lesions in a patient with Chikungunya Fever before **(a–c)** and after **(d–l)** intravenous treatment with intravenous immunoglobulin IVIG-Flebogamma®.

We report a case of a 56-year-old man presenting acute fever, cutaneous rash, conjunctival hyperemia, intense joint pain, and self-reported use of non-steroidal anti-inflammatory drugs (NSAID) in the initial days of symptoms. The patient reported that for the last 30 days before Hospital admission, he started presenting fever, headaches, paresthesia, and pain in the right arm with visible red spots on the skin. These skin lesions worsened and spread through the lower limbs and trunk within a period of 10 days. The patient evolved to hypotension with some Hospital admissions and discharges. On the 15th day after skin disease onset, he developed thrombocytopenia, liver dysfunction with International Normalized Ratio (INR) of 1.45 and Prothrombin Time of 56%, edema in his hands and feet and hemorrhagic bullous lesions on the skin of the upper and lower limbs (Figures 1a–c), being admitted to the Intensive Care Unit. Immediately, therapy was started with meropenem and vancomycin, then maintained for 6 days, during which the patient presented some febrile peaks. Subsequently, intravenous administration of Intravenous Immunoglobulin (Human), 5% (Flebogamma® 5% DIF, Instituto Grifols S.A., Barcelona, Spain) at 400 mg/Kg/day restarted for 5 days. Antibiotic therapy started again for 5 days. The patient showed a progressive increase in platelet levels from 43,000 to 201,000 and total leukocyte count, together with an important reductions of the edema, necrosis, and erythema. Ten days after globulin administration, a substantial improvement of the bullous lesions was observed (Figures 1g–l). The patient evolved with aphasia, thus being considered to be suffering a transient acute ischemic stroke. Laboratory analysis followed the Pan American Health Organization (PAHO) recommendations, in which a single anti-CHIKV IgM-positive test (collected during acute or convalescence phase) is sufficient for confirmation of any suspected case of CHIKV (15). The blood test returned positive serology for Dengue (IgM^−^/IgG^+^) and Chikungunya (IgM^+^) at the 16th day after symptom onset, but results were negative for other infectious diseases and blood culture. Notably, laboratory tests excluded other confounding diseases such as: malaria, leptospirosis, rheumatic fever, septic arthritis, Zika, and Mayaro. Moreover, there is no report showing any Mayaro case detected in the state of Paraíba. This would definitively exclude the possibility of a cross-reactive IgM serology against another alphavirus as discussed elsewhere (16). No comorbidities were reported, except for alcoholism. Throughout hospitalization, imaging exams were normal and no vasoactive drugs were administered. Furthermore, no lesions were observed in oral, genital, or conjunctival mucosa. Nikolsky's sign was positive. A diagnosis of Stevens Johnson syndrome-Toxic epidermal necrolysis-like features was discarded. The patient was discharged and has not presented any other clinical sign of the disease.

## Discussion

Since the latest epidemics, a considerable increase in mortality rate of CHIKV-infected patients has been observed in a 2005 outbreak that occurred on the island of Réunion in the Indian Ocean (3). Mortality was often associated with neurological damage affecting neonates, immunocompromised patents and the elderly (11, 17–19). The occurrence of bullous lesions on the skin of children with severe forms of CHIKV infection was strongly associated with higher viremia and lethality rates (20). In adults, however this association still needs clarification. Some reports in India found skin rash in suspected patients during the acute phase of the illness presented (7, 21–23). Desquamation was observed on palms, soles and face in about 10% of probable CHIKV-infected patients, lasting up to 2 weeks after disease onset (22).

The patient herein presented an atypical form of CHIKV infection with extensive skin lesions, requiring ICU admission. One possible explanation for this complicated CHIKV case would be a previous dengue virus infection demonstrated by IgM^−^/IgG^+^ serology. Although speculative, some authors hypothesize that a previous infection with one arbovirus would be responsible for a potentiation of the antibody-dependent response in a novel infection in endemic areas with overlapping arbovirus transmission (24). Another possible explanation would be that a virulent CHIKV strain would be circulating preferentially in this region of the country. For clarification of these two speculations, larger studies are needed. In relation to the patient's bullous skin lesions, differential diagnosis of Stevens Johnson syndrome-Toxic epidermal necrolysis-like features diagnosis was discarded. Lesions were highly characteristic and well described in the literature as a consequence of CHIKV infection in severe and atypical forms of the disease (25, 26). Moreover, patient self-reported alcoholism reinforces the idea that this behavior might be considered an important risk factor for atypical and severe forms of CHIKV infection (4, 27).

In relation to the disease immunopathology, experimental models of CHIKV infection demonstrated that viral replication induces two patterns of physiological damage, with increased cell death, intense cytokine production, and tissue edema at 2–3 days post-infection. Subsequently, 6–7 days after infection, the second round of pathophysiological events might be associated with virus clearance and infiltration of huge inflammatory cells into joints (28, 29). The activation of monocytes/macrophages and neutrophils within tissues seems to be determinants of the inflammatory damage observed in CHIKV infection. Contrarily, protective responses are dependent on type I IFNs and TLR3 signaling pathways in innate cells, whereas CD4^+^ T cell-dependent production of neutralizing antibodies by B cells would be the key mechanism in humans (30). In humans, again, severe CHIKV infection has been associated with elevated levels of pro-inflammatory biomarkers, mostly Th1 cytokines and chemokines, in both innate and adaptive immune response pathways (30, 31). In addition to that observed in mice, activation of monocytes/macrophages and NK cells were encountered in biopsies obtained from chronically infected non-human primates and in chronic human patients, which suggested the potential role of these cells in the extensive inflammatory damage of the tissues (32, 33). More evidence on the role of these cells as targets for CHIKV infection and tissue damage has been associated with elevated production of chemokine CCL2/MCP-1 during the acute phase of the disease in humans and in animal models (31, 32, 34). In human chronic infection as well as in non-human primates, CHIKV might be detected by immunohistochemistry especially in tissue macrophages (31, 33). Moreover, the virus strongly activates NK cells and osteoclasts, which induces a generalized inflammatory response even in the chronic period of the infection (35). Thus, strategies (e.g., IVIG or anti-CHIKV immunoglobulin-based therapies) aimed at controlling the pro-inflammatory behavior of monocytes/macrophages in the very early period of the infection might be beneficial to the patients. Still, the use of immunoglobulins *in vivo* to treat CHIKV infected patients is poorly reported in the literature.

The lack of specific treatments for severe and atypical forms of chikungunya fever hinders clinicians to manage these patients. Previously, the usefulness of immunoglobulins purified from CHIKV-infected patients was reported in mouse models of CHIKV infection (9). This finding suggested that the administration of human antibodies to treat CHIKV infection would be an alternative therapy. Although the currently proposed treatment does not include anti-CHIKV monoclonal antibodies, the potential function of the immunoglobulins present in the commercial IVIG-Flebogamma^®^ pool would be beneficial to the patient by providing a systemic anti-inflammatory response (36–38). Although the exact mechanism involved in the theoretical anti-inflammatory effect observed in the CHIKV patient needs elucidation, the immunomodulatory mechanisms of IVIG treatment determined in numerous hematological, rheumatological, neurological, and dermatological disorders might be occurring in the successful outcome of CHIKV-infected patients receiving this treatment. Thus, IVIG has been considered to have four different mechanistic components that may be acting independently or concurrently in different settings (39). In relation to the first mechanism, it has been proposed that variable regions F(ab′)2 would mediate binding site interactions with many components promoting anti-proliferative effects, modulation of apoptosis and cell cycle, activation of specific cells, interference in cell adhesion, induction of antibodies directed to pathogens, superantigens, to immunoregulatory molecules (i.e., TCR and CD4) and to cytokines (40). IVIG contains antibodies specific to a broad range of pathogens, reflecting the antibody repertoire of the thousands of donors included in each batch of commercial preparation. Anti-staphylococcal superantigen antibodies present in IVIG formulation were able to inhibit superantigen-mediated activation of T cells, which might be its key immunomodulatory mechanism in disorders such as Kawasaki disease and atopic dermatitis. However, antibody levels against specific pathogens can vary enormously between batches (41). Natural antibodies against interleukin-1α (IL-1α), IL-8 and tumor necrosis factor-α (TNF-α) have been titrated in the sera of healthy individuals. Furthermore, assessment of intracellular cytokine production by mitogen-stimulated PBMCs in the presence of IVIG showed strong reductions in IL-3, IL-4, IL-5, TNF-β, and granulocyte–macrophage colony-stimulating factor (GM-CSF) producing cells (42, 43). Also, the higher-order aggregates of IgG in IVIG preparations have been shown to activate neutrophils via triggering of macrophages (44).

In its second action mechanism, the IVIG pool exhibits potent effects on Fc receptors, which might inhibit phagocytosis, and antibody-dependent cell cytotoxicity (ADCC) as well as antibody production and recycling and glucocorticoid receptor binding affinity (39). The beneficial effects of IVIG administration in immune cytopenias appear to be a result of the saturation of FcR on splenic macrophages by massive binding of the Fc portion of administered immunoglobulins (45). Moreover, IVIG administration might induce the surface expression of FcγRIIB on splenic macrophages. Modulation of inhibitory (FcγRIIB) signaling is a potent mechanism for attenuating autoantibody-triggered inflammatory diseases and infectious diseases by inhibiting the activation of B lymphocytes, monocytes, mast cells, and basophils induced by activating receptors (46). The third immunomodulatory mechanism would be mediated by complement binding to the Fc fragment present in IVIG pools. This can interrupt the assembly and deposition of the complement fragments forming the membrane attack complexes (MAC) on the endomysial capillaries, while complexes between IVIG antibody and C3b fragment are formed. This would prevent the incorporation of activated C3 into C5 convertase. MAC deposition onto the intramuscular capillaries is one of the mechanisms underlying the pathogenesis of dermatomyositis. This is inhibited by IVIG treatment and would explain the successful use of this therapy in pemphigus and mucous membrane pemphigoid dermatological diseases (14, 47). The fourth mechanism would be the presence of immunomodulatory substances other than antibodies in the IVIG preparations, such as cytokines, cytokine receptors, MHC class II and sugars functioning as stabilizing agents that can inhibit many proliferative response (48). Recently, it has been demonstrated that IVIG can decrease the expression of surface markers including class II HLA and co-stimulation molecules such as CD80^+^, CD86^+^, and CD40^+^ as well as many cytokines produced by dendritic cells. Also, IVIG infusions may influence the Th17/Treg balance and enhance immune tolerance (49). Consequently, this therapy would be involved in both FcR-dependent and independent mechanisms of granulocyte cell death (38). These effective mechanisms might also account for the successful results obtained by previous reports of CHIKV-associated neurological symptoms (11, 12) and in a case series of patients with Guillain–Barré syndrome (13). The relationship between the inflammatory status induced by CHIKV infection and disease progression has been explored by many studies on human and animal models of the disease. Therefore, the observed immunomodulatory effects of IVIG seems to depend on both the dose used (replacement or high dose) and the disease being investigated. Until nowadays, the exact immunomodulatory mechanisms induced by IVIG treatment in Chikungunya fever are unclear. Its interaction with many arms of the immune system might help explain its effectiveness in treating CHIKV-infected patients that present atypical dermatological manifestation. For the first time, it is reported herein that this strategy can treat the atypical dermal involvement of the disease with successful recovery of the patient.

## Conclusion

The unique results reported herein, together with the literature data, suggest that the use of polyvalent globulins, and to an extent the development of commercial anti-CHIKV-specific neutralizing antibodies, would constitute an excellent, and safe option for treating CHIKV-infected patients presenting atypical dermatological forms of the disease.

## Ethics Statement

This work was part of a larger project approved by the UFPB Ethical Committee on Human Research (protocol #032/2009/CEP/HULW/UFPB). Written informed consent was obtained from the patient for publication of this case report.

## Author Contributions

AF, AS, and SC were involved in patient management, diagnosis, literature review, and drafting of the manuscript. JS, SC, and LC were involved in laboratory diagnosis. AF, JS, and LC critically reviewed the manuscript. All authors read and approved the final version of the manuscript.

### Conflict of Interest Statement

The authors declare that the research was conducted in the absence of any commercial or financial relationships that could be construed as a potential conflict of interest.
